# Quality and Accessibility of Home Assessment mHealth Apps for Community Living: Systematic Review

**DOI:** 10.2196/52996

**Published:** 2024-03-11

**Authors:** Jung-hye Shin, Rachael Shields, Jenny Lee, Zachary Skrove, Ross Tredinnick, Kevin Ponto, Beth Fields

**Affiliations:** 1 Department of Design Studies University of Wisconsin-Madison Madison, WI United States; 2 Department of Kinesiology University of Wisconsin-Madison Madison, WI United States; 3 Wisconsin Institute for Discovery University of Wisconsin-Madison Madison, WI United States

**Keywords:** mobile app, mobile applications, mHealth, mobile health, app, apps, application, applications, mobile phone, mobile app rating system, occupational therapy, home assessment, web accessibility, aging in place, accessible, accessibility, quality, rating, gerontology, geriatric, geriatrics, older adult, older adults, elder, elderly, older person, older people, ageing, aging, systematic, synthesis, syntheses, PRISMA, Google Play, content analysis, functionality, WCAG

## Abstract

**Background:**

Home assessment is a critical component of successful home modifications, enabling individuals with functional limitations to age in place comfortably. A high-quality home assessment tool should facilitate a valid and reliable assessment involving health care and housing professionals, while also engaging and empowering consumers and their caregivers who may be dealing with multiple functional limitations. Unlike traditional paper-and-pencil assessments, which require extensive training and expert knowledge and can be alienating to consumers, mobile health (mHealth) apps have the potential to engage all parties involved, empowering and activating consumers to take action. However, little is known about which apps contain all the necessary functionality, quality appraisal, and accessibility.

**Objective:**

This study aimed to assess the functionality, overall quality, and accessibility of mHealth home assessment apps.

**Methods:**

mHealth apps enabling home assessment for aging in place were identified through a comprehensive search of scholarly articles, the Apple (iOS) and Google Play (Android) stores in the United States, and fnd.io. The search was conducted between November 2022 and January 2023 following a method adapted from PRISMA (Preferred Reporting Items for Systematic Reviews and Meta-Analyses). Reviewers performed a content analysis of the mobile app features to evaluate their functionality, overall quality, and accessibility. The functionality assessment used a home assessment component matrix specifically developed for this study. For overall quality, the Mobile Application Rating Scale (MARS) was used to determine the apps’ effectiveness in engaging and activating consumers and their caregivers. Accessibility was assessed using the Web Content Accessibility Guidelines (WCAG) 2.1 (A and AA levels). These 3 assessments were synthesized and visualized to provide a comprehensive evaluation.

**Results:**

A total of 698 apps were initially identified. After further screening, only 6 apps remained. Our review revealed that none of the apps used thoroughly tested assessment tools, offered all the functionality required for reliable home assessment, achieved the “good” quality threshold as measured by the MARS, or met the accessibility criteria when evaluated against WCAG 2.1. However, DIYModify received the highest scores in both the overall quality and accessibility assessments. The MapIt apps also showed significant potential due to their ability to measure the 3D environment and the inclusion of a desktop version that extends the app’s functionality.

**Conclusions:**

Our review revealed that there are very few apps available within the United States that possess the necessary functionality, engaging qualities, and accessibility to effectively activate consumers and their caregivers for successful home modification. Future app development should prioritize the integration of reliable and thoroughly tested assessment tools as the foundation of the development process. Furthermore, efforts should be made to enhance the overall quality and accessibility of these apps to better engage and empower consumers to take necessary actions to age in place.

## Introduction

Home modifications are essential procedures for individuals with various functional limitations, enabling them to live independently within their own community. Traditionally seen as targeted biopsychosocial interventions, these modifications aim to address the functional limitations experienced by aging adults and individuals living with disabilities in their home environments. Additionally, home modifications are frequently used as part of hospital discharge planning after medical treatments such as geriatric and stroke rehabilitation [1,2]. Conducting a timely home assessment using a valid instrument and promptly implementing home modifications is crucial in assisting individuals recovering from medical procedures. These steps help maintain their functional abilities and ensure a reasonable quality of life in their homes [1,3,4].

The current gold standard for home modifications necessitates a systematic home assessment conducted by trained professionals, often performed by occupational therapists, as a prerequisite [3]. However, accessing such services remains challenging for many consumers [2,5,6]. Several contributing factors include (1) the lack of standardized and validated assessments and limited knowledge of best practices [7]; (2) the shortage of professionals trained to conduct these assessments, particularly in rural areas [6,8-10]; (3) the consumer’s perceived burden from participating in comprehensive assessments [11,12]; and (4) the complexity and cost involved in conducting home assessments [13-16].

Reliable and validated home assessment tools do exist, albeit in a paper-and-pencil format. In a systematic review of the psychometric properties of available home accessibility assessment tools, Patry et al [7] identified several tools that meet critical psychometric properties, including In-Home Occupational Performance Evaluation (I-HOPE) [17], I-HOPE Assist [18], Housing Enabler (HE) [19,20], Comprehensive Assessment and Solution Process for Aging Residents [21], and Home and Community Environment [22]. However, challenges still exist in using these tools, such as the laborious and time-consuming measurement process and the difficulty in getting reimbursement for the cost of an occupational therapist’s time [23]. The lack of objective environmental measurement has also been identified as a weakness of these tools, except for the HE [24].

The cost barrier and limitations in objectively measuring the physical environment have prompted researchers to explore the use of teleconferencing for remote home assessment tools [8,9,25]. More recently, several entities have started developing mobile health (mHealth) apps that integrate 3D modeling [26], virtual reality [27], and augmented reality (AR) tools [28,29] to measure, store, and share spatial data required for home modification solutions. However, what remains less understood and documented are the functionality, quality, and accessibility of mHealth apps. This is problematic as the number of mHealth apps for home assessment continues to increase, and there is no available evidence-informed guidance on which ones to use and why.

Therefore, the objective of this study was to systematically identify and evaluate publicly available mHealth apps available in the United States that focus on home modification in the context of aging in place, using three important criteria: (1) comprehensiveness of functionality, (2) overall quality leading to consumer engagement and follow-up actions, and (3) accessibility. A well-developed tool with all necessary functions can help professionals and consumers perceive that the app possesses the features and qualities they need to support collaboration with home modification providers in achieving desired goals [10,30].

## Methods

### App Identification

The research team conducted a systematic search across multiple information sources, including a database search of peer-reviewed journal papers on home assessment, the Apple (iOS) and Google Play (Android) stores in the United States, and fnd.io. The team followed PRISMA (Preferred Reporting Items for Systematic Reviews and Meta-Analyses [31]) guidelines whenever applicable (Multimedia Appendices 1 and 2) and referred to reviews focused on mHealth apps [32-34]. The initial search was conducted between November 2022 and January 2023.

The first author (JS) conducted a search of 4 databases, including Academic Search Premier, APA PsycInfo, Consumer Health Complete—EBSCOhost, and MEDLINE. The search terms used were (“home assessment” or “home modifications” or “home mods” or “home adaptations”) AND “occupational therapy” AND (technology or application or computer or tablet or mobile phone or smartphone or internet). Titles and abstracts were then reviewed using the following criteria:

- Years considered: 1990-2022
- Language: English
- Publication status: Published
- Publication type: Includes articles in peer-reviewed journals, encompassing all types of publications
- Home assessment focus: Accessibility, covering parts of or the entire house
- Types of functional limitations: All forms of functional limitations, including both physical and mental
- Exclusion criteria: Gaming apps for occupational therapy or medical training and exclusive use of technology for communication (telehealth)

In addition to articles on individual apps, the database search yielded 3 recent research publications conducting a meta-analysis of home assessment tools [10,23,35], prompting further manual searches.

The app store search was carried out by JL. JL systematically conducted individual searches on the Google Play store using a Samsung Galaxy S21 phone and on the Apple App Store using an iPhone 11. The search terms used were consistent with those used in the database search. To ensure comprehensive coverage of potentially relevant apps, the same method was applied to searches on the fnd.io website by the author RS.

All 3 reviewers (JS, RS, and JL) convened to establish common exclusion criteria and reach consensus on the final list of apps for analysis. The exclusion criteria encompassed (1) apps not intended for home assessment; (2) unstable operations that impeded effective use, such as frequent crashes and errors; (3) regional restrictions that limited access to certain apps for US users; and (4) apps with dubious objectives, such as prioritizing product promotion over facilitating home modifications for enhanced accessibility.

### Data Extraction

Between January 2023 and March 2023, three distinct tools were used to evaluate the quality of the chosen apps: an app component matrix developed for this study, the Mobile App Rating Scale (MARS) [36], and the Web Content Accessibility Guidelines (WCAG) 2.1 (created by the World Wide Web Consortium) [37].

The analysis of app components focused on evaluating the features, capabilities, and operations of each home assessment tool to understand its usefulness compared with traditional paper-and-pencil evaluations [23]. The primary objective of this evaluation was to assess the potential of each app to be effectively used by professionals or consumers in the field. Traditional and validated home assessment tools, such as I-HOPE [17] and HE [38,39], typically enable evaluators to assess the functional limitations of residents and evaluate the physical aspects of the home environment to identify necessary adjustments for the identified functional limitations. These assessments typically do not encompass suggestions for subsequent home modifications, as such considerations lie beyond the purview of home assessments. Nonetheless, given that a significant number of the reviewed apps featured recommendations, the incorporation of recommendations was introduced into the component matrix for this study. Overall, the examination centered on assessing whether each app empowers users to appraise functional limitations, conduct home environment assessments (through checklists or measurements), generate assessment outcome reports, and offer recommendations.

The MARS is a reliable and objective tool used for classifying and assessing the overall quality of mHealth apps [36]. Unlike star ratings in app stores or subjective app reviews, the MARS provides a systematic approach to evaluate mHealth apps, offering a more comprehensive and reliable measure of their quality. Studies conducted by Stoyanov et al [36] and Terhorst et al [40] have reported a high level of construct and concurrent validity, as well as reliability and objectivity, with an intraclass correlation coefficient ranging between 0.82 and 0.85. This indicates a strong level of consistency among different MARS raters, further highlighting the reliability of the scale. Moreover, the MARS has been used by researchers to assess the ability of mHealth apps to engage and activate patients [34].

The MARS consists of 3 main components: App Quality Questions, App Subjective Quality Questions, and App Specific Questions. The App Quality Questions cover various categories to provide a comprehensive evaluation of the app. These categories include engagement (A), functionality (B), aesthetics (C), and information quality (D). The engagement category assesses factors such as fun, interest, individual adaptability, interactivity, and target group. Functionality focuses on the app’s performance, usability, navigation, and gestural design. Aesthetics evaluates the layout, graphics, and visual appeal of the app. Information quality examines the accuracy, quantity, and quality of information provided, including the credibility and evidence base of the app.

In addition to the App Quality Questions, the MARS includes App Subjective Quality Questions to capture the reviewer’s personal opinion and the perceived impact on the user. The reviewer’s personal opinion (E) covers aspects such as app recommendation, willingness to pay for it, anticipated frequency of usage, and an assigned star rating. The perceived impact on the user (F) assesses how the app affects the user’s knowledge, attitudes, intentions to change, and the likelihood of actual change in the target health behavior.

Each question in the MARS is aligned with a 5-point scale (1=inadequate, 2=poor, 3=acceptable, 4=good, 5=excellent). This scale provides a standardized framework for rating the app’s performance across different categories. The unique structure and scale of the MARS allow reviewers to holistically evaluate mobile apps, considering both objective quality indicators and subjective assessments.

The research team also used the WCAG 2.1 [37]. These guidelines are designed to make web content more accessible to all users but is highly relevant to web and nonweb mobile phone content [41]. Considering that the target population of home assessment apps includes people with functional limitations, ensuring accessibility is deemed critical for the successful deployment of these apps. In comparison with the MARS rating, which primarily provides a general evaluation of mHealth apps, the WCAG assessment offers a comprehensive framework for evaluating accessibility criteria.

The WCAG 2.1 has four criteria categories: (1) perceivable, (2) operable, (3) understandable, and (4) robust. The “perceivable” category highlights the importance of users being able to perceive all presented information using their available senses. “Operable” refers to an interface that is easily navigable and usable by a wide range of users. The “understandable” category includes criteria ensuring that users should have no difficulty comprehending both the content and the user interface. Lastly, “robust” ensures that the content is consistently and accurately interpreted by a diverse range of user agents, including assistive technologies.

WCAG 2.0 was initially released in December 2008 and was adopted into Section 508 of the Rehabilitation Act (29 USC 794d) in 2018. Any project receiving federal funds must adhere to Section 508 of the Rehabilitation Act. WCAG has 3 conformance levels: A, AA, and AAA, with level AAA being the highest level. To understand how WCAG operates, it is crucial to recognize that content meeting a higher level of compliance also satisfies all the criteria of a lower level. Additionally, it is worth highlighting that achieving full compliance with high-level standards is uncommon among apps, mainly due to the inherent difficulties and resource-intensive nature involved in meeting these criteria. This study used both the A and AA levels of WCAG 2.1 to evaluate the accessibility of all identified apps.

### Data Analysis

The identified mHealth apps underwent testing and assessment by 3 reviewers (RS, JL, and ZS), with each being evaluated one at a time. All apps were installed and tested using an iPhone 13. The diverse training backgrounds of the 3 reviewers represented a mix of professionals likely to use or be involved in app development. ZS brings 2 years of experience in occupational therapy. RS has training and professional experience in architecture and landscape architecture, with substantial knowledge of building accessibility. JL’s training encompasses interior design, user interface design, and graphic design expertise; she also has substantial knowledge of building accessibility.

The reviewers individually examined each app and determined if it included assessment components commonly found in traditional paper-and-pencil home assessment tools: functional limitations, physical environment assessment area, mode of measurement (checklist vs measurement), final report, and recommendations. As they evaluated these components, they considered observations during app usage and information from the app store. The components were agreed on during a postassessment meeting before being included in a matrix.

The MARS rating procedures followed the recommendations specified in the original study [36]. All 3 reviewers familiarized themselves with the MARS and watched the training video to gain a thorough understanding of its components and dimensions. Before assessing apps, they engaged in extensive discussion and consensus building to clarify the meaning and relevance of each MARS assessment item.

Subsequently, the reviewers extensively used each selected app for review to gain a comprehensive understanding of its features, functionality, content, and user experience. After individually assessing the apps, the reviewers convened to reach a consensus on the final scores and tabulated the mean scores. The mean scores were compiled from each section of the MARS, namely, (A) engagement, (B) functionality, (C) aesthetics, and (D) information. The app quality mean score was calculated by averaging the scores from these 4 sections. App subjective quality (E) and app-specific (F) scores were separately averaged and treated as distinct measures. The questions in the (F) section, which evaluate the perceived impact of the app on the user’s knowledge, attitudes, intentions to change, and the likelihood of actual change in the target health behavior, were specifically responded to considering the target behavior of home assessment and modification, aligning them with the scope of this study. The agreed-on ratings were then compiled into a matrix to facilitate comparisons and analysis of ratings and data across the apps.

The accessibility assessment involved checking apps against all WCAG 2.1 criteria. During a preassessment meeting, the reviewers engaged in a detailed discussion of each of the 50 WCAG criteria. They collaboratively developed concise descriptions in the newly created WCAG evaluation form (Multimedia Appendix 3) and established a consensus on how to assess the apps based on these criteria. Individually, the reviewers assigned a pass, fail, or NA (not applicable) designation to each of the 50 WCAG criteria. The NA designation was used when a particular criterion covered a function that was not present in the app being evaluated.

In the postassessment meeting for each app, the reviewers convened to deliberate on the designations for each WCAG criterion and worked together to reach a consensus. Subsequently, the passing percentage for each WCAG category (perceivable, operable, understandable, and robust) was calculated, along with an overall passing percentage. The passing percentages were determined by excluding any criteria assigned an NA designation from the calculation.

The results of the quality and accessibility appraisal were visualized in a map. The map represents the quality appraisal using the MARS on the horizontal axis and WCAG 2.1 on the vertical axis. To enhance the objectivity of the MARS as a measure of app quality [36], we used the average score from the objective MARS items on the horizontal axis. On the vertical axis, we considered 2 levels of WCAG 2.1, namely, A and AA. Apps that scored higher on both parameters indicate high quality and accessibility, suggesting a greater potential for user engagement and activation [34,36].

## Results

### Identification

The initial database search yielded 104 articles, which were then reduced to 36 after eliminating duplicates. Among these, 3 meta-analyses on home assessment tools were included. After a comprehensive review of all identified articles from the database search, 3 mHealth apps were identified: HESTIA, MapIt Mobile, and Magicplan. After an initial assessment of downloadable apps, HESTIA was excluded due to its ongoing development and unavailability for download.

On engaging with the development team of MapIt, it was discovered that the desktop version of the app offered significantly enhanced functionality compared with its mobile counterpart. Because of the distinct interfaces between the 2 versions, separate evaluations were conducted for each. Although MapIt Desktop is not classified as an mHealth app, it was included in the evaluation due to its close association with the mobile app and its robustness in offering 3D measurement capabilities in the context of home assessment, which were not found in any other apps.

The initial app store search yielded a substantial 690 apps. After reviewing titles, descriptions, preview photos, and keywords, 23 apps remained. The fnd.io search yielded a total of 253 apps, which were then narrowed down to 16 apps using the same screening method used for the app store search.

**Figure 1:**
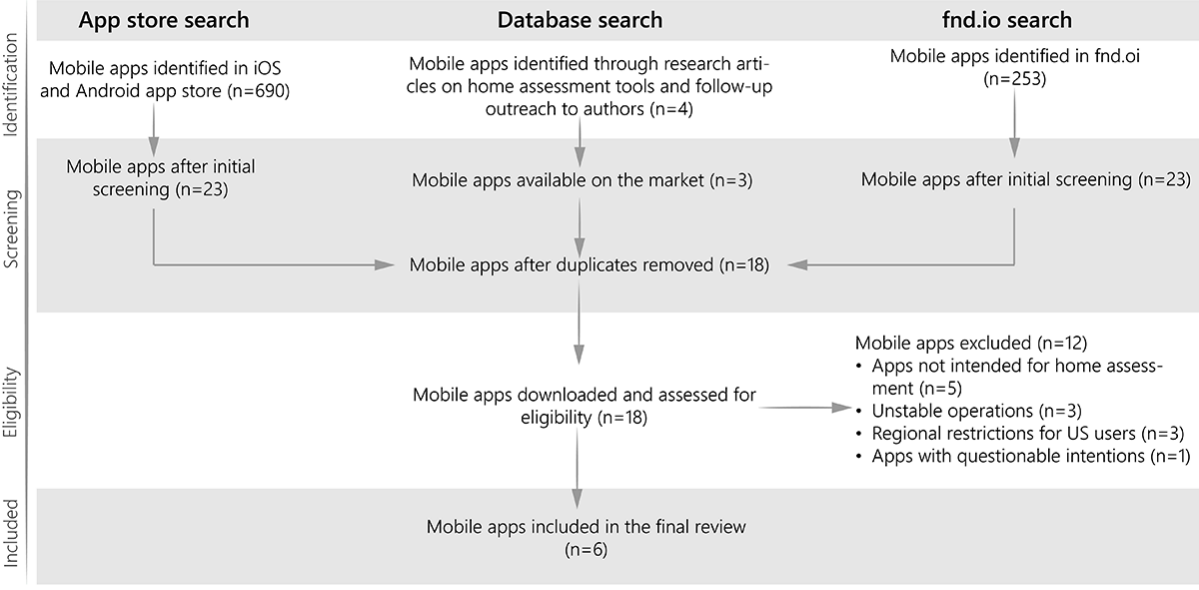
Identification process of mobile health apps for home assessment in the United States.

After removing duplicates from all 3 sources, 18 apps remained, all of which were downloaded for eligibility evaluation. Eventually, 12 apps were eliminated due to exclusion criteria, leaving 6 apps for detailed analysis: BEAT-D, DC Carehomes, DIYModify, HomeFit AR, MapIt Mobile, and MapIt Desktop (Figure 1).

### Characteristics of the Included Apps

The characteristics of the included apps are presented in Table 1. Of the 6 apps reviewed, 4 (67%) apps were developed in university settings, 1 (17%) app was developed by a private company, and 1 app was developed by AARP, a nonprofit organization in the United States. Geographically, 3 (50%) apps (BEAT-D, DC Carehomes, and DIYModify) were developed in Australia, whereas 2 (33%) apps (MapIt Mobile and MapIt Desktop) were developed in Canada. The latter 2 apps were developed by the same entity, and they are functionally complementary to each other. Of the 6 apps, 1 (17%) app (HomeFit AR) was developed in the United States.

All the apps were designed for the iOS environment. Moreover, DC Carehomes and DIYModify were also developed for the Android platform. All apps were free to download. None of the reviewed apps were categorized as medical products nor had they published trials evaluating the effectiveness of the apps.

Among the 6 apps, only 2 (33%) apps (MapIt Mobile and MapIt Desktop) allowed the users to take actual measurements of the environment. These apps provided a feature for users to gather specific measurements. In contrast, all other apps were questionnaire-based interactive decision-making tools that asked a series of questions to the users as part of the home assessment process (Tables 1 and 2).

**Table 1:** Operating characteristics and summary of identified mobile health apps.

App name	Logo	Platform and operating system	Developer	Assessment characteristics: app summaries
BEAT-D	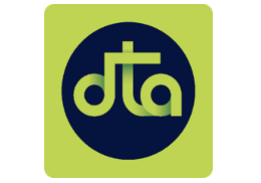	Mobile, tablet, and iOS	University of Wollongong, Australia	Questionnaire: a guided questionnaire assessment for buildings designed to accommodate individuals with dementia. The user’s responses are compiled into a comprehensive report, which identifies areas that need improvement in order to reduce confusion, agitation, and depression.
DC Carehomes	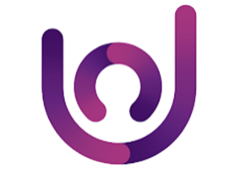	Mobile, web, tablet, iOS, and Android	Private Company: Hammond Care, Australia	Questionnaire: a guided questionnaire assessment for care homes, units, or households catering specifically to individuals with dementia. The app generates a comprehensive report that offers recommendations based on the assessment findings.
DIYModify	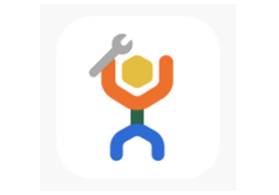	Mobile, iOS	University of New South Wales, Australia	Questionnaire: an interactive decision-making tool that concentrates on 5 particular home modifications and provides guidance. It helps users select appropriate product types that match their needs and offers instructions on taking necessary measurements before shopping for home modifications. The app includes real-life stories of individuals who have undergone these specific adaptations, allowing users to learn from their experiences.
HomeFit	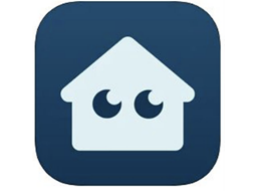	Mobile, tablet, and iOS	Nonprofit: AARP, United States	Questionnaire: an interactive decision-making tool assists users in identifying potential home improvements for aging in place. The app generates a comprehensive report, including tips, suggestions, and a checklist, based on the user’s responses. The checklist distinguishes between tasks suitable for do-it-yourself and those requiring professional assistance. While the app uses AR^a^ to recognize specific features such as a kitchen sink, it does not use AR for actual measurement purposes.
MapIt	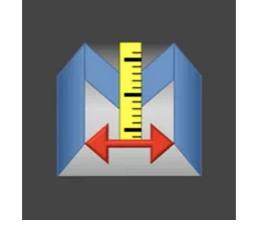	Mobile, tablet, iOS, and Android	University of Sherbrooke, Canada	Measurement: the app uses AR and the LiDAR^b^ sensor on the phone to create a 3D scan of a room. Users can then add measurements to specific areas of interest within the scan, catering to accessibility needs. The scan can be exported and viewed in the MapIt Desktop version, enhancing the overall measurement experience.
MapIt Desktop	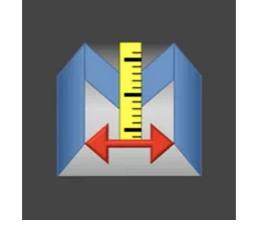	Windows desktop, Mac OS X, and Windows	University of Sherbrooke, Canada	Measurement: this desktop app leverages MapIt on an iPhone to capture 3D scans of rooms, which can subsequently be imported to facilitate space measurements.

^a^AR: augmented reality.

^b^LiDAR: Light Detection and Ranging.

**Table 2:** Functional components and mode of assessment of the reviewed apps.

App name	Functional assessment	Environmental assessment				Summary report	Recommendation	Mode of assessment
		Entrance	Bathroom	Kitchen	Bedroom			
BEAT-D	—^a^	N^b^	N	N	N	Yes	Yes	Checklist
DC Carehomes	—	N	N	N	N	Yes	Yes	Checklist
DIYModify	—	T^c^	T	—	—	—	Yes	Checklist
HomeFit	—	—	T	T	T	Yes	Yes	Checklist
MapIt Mobile	—	Generic	Generic	Generic	Generic	—	—	Measurement
MapIt Desktop	—	Generic	Generic	Generic	Generic	—	—	Measurement

^a^Not available.

^b^N: nontargeted.

^c^T: targeted.

### Component Analysis of the Included Apps

Table 2 includes a summary of the component analysis. Among the 6 apps assessed, none of them considered the functional limitations of the resident. Consequently, none of the apps enabled the evaluation of the physical environment tailored to the resident’s individual functional capacities.

DIYModify and HomeFit AR stood out by delivering concentrated assessments for certain critical spaces, notably the entrance, bathroom, kitchen, and bedroom, designated as “targeted” (T) in Table 2. DIYModify, in particular, enabled users to assess essential elements within entrance and bathroom areas, while HomeFit emphasized assessment of the bathroom, kitchen, and bedroom areas. However, it is worth noting that no single app assessed all of the key areas comprehensively. Conversely, the remaining apps either lacked specific evaluations for the designated target spaces—although they did include questions related to those areas, classified as “nontargeted” (N)—or presented generalized assessment tools adaptable to any area, categorized as “Generic” in Table 2.

The reporting modules within these apps should ideally furnish a concise overview of assessment findings on each evaluation’s conclusion, aiding users in comprehending the assessment outcomes and strategizing potential home modifications. Among the 6 apps under scrutiny, only HomeFit, DC Carehomes, and BEAT-D yielded a comprehensive report after completion of the assessment. Notably, HomeFit AR, DIYModify, DC Carehomes, and BEAT-D offered recommendations. Conversely, DIYModify omitted the provision of a report, whereas both MapIt Mobile and Desktop were deficient in both report and recommendation functionalities.

### Quality Appraisal of the Included Apps: MARS

The MARS scores for the 6 parameters of (A) engagement, (B) functionality, (C) aesthetics, (D) information, (E) subjective rating of the app overall, and (F) subjective ratings of app-specific features are presented in Table 3. Very few apps received a rating of “good” (4 or above [36]) across the measured parameters, although many of them achieved an “acceptable” (3 or above and below 4) range.

**Table 3:** Mobile Application Rating Scale (MARS) objective and subjective quality criteria and the assessment result.

App name	Objective quality					Subjective quality		
	(A) engagement	(B) functionality	(C) aesthetics	(D) information	Overall objective quality	(E) app overall	(F) app specific	Overall subjective quality
BEAT-D	1.6	3.75	3.3	3.4	3.0	2	3.0	2.5
DC Carehomes	2	3.5	3.3	4^a^	3.2	2	3.2	2.6
DIYModify	3	3.75	4^a^	4^a^	3.7	3.5	4.7^a^	4.1^a^
HomeFit AR	2.8	3	3.7	2.83	3.1	1.75	2.7	2.2
MapIt Mobile	3	2.75	3	3.25	3.0	3	1.5	2.3
MapIt Desktop	3.2	3.5	3.7	3.25	3.4	3.5	2.5	3

^a^Apps considered “good” (4 or above) under subcategories of the MARS assessment criteria.

When using the MARS objective quality criteria, none of the apps were rated as good in the categories of (A) engagement and (B) functionality. In the (C) aesthetic category, only DIYModify achieved a good rating with a score of 4. In the (D) information category, 2 apps, namely DC Carehomes and DIYModify, scored 4 and were thus classified as good. However, when considering the overall objective quality of the apps (mean of A, B, C, and D), none of the apps reached the threshold of 4.

Ratings for the MARS subjective quality were even lower. None of the reviewed apps reached the threshold of “good” in the (E) subjective rating of the app overall, and only half of them reached an “acceptable” level. Apps performed similarly in (F) subjective ratings on the app-specific features. However, DIYModify achieved an exceptionally high score of 4.7 out of 5. The incorporation of real-life video stories showcasing practical and effective home modifications within the app contributed to the high score, as it significantly enhanced the app’s potential to positively influence the user’s knowledge, attitudes, and actual behavior change in relation to home assessment.

**Figure 2:**
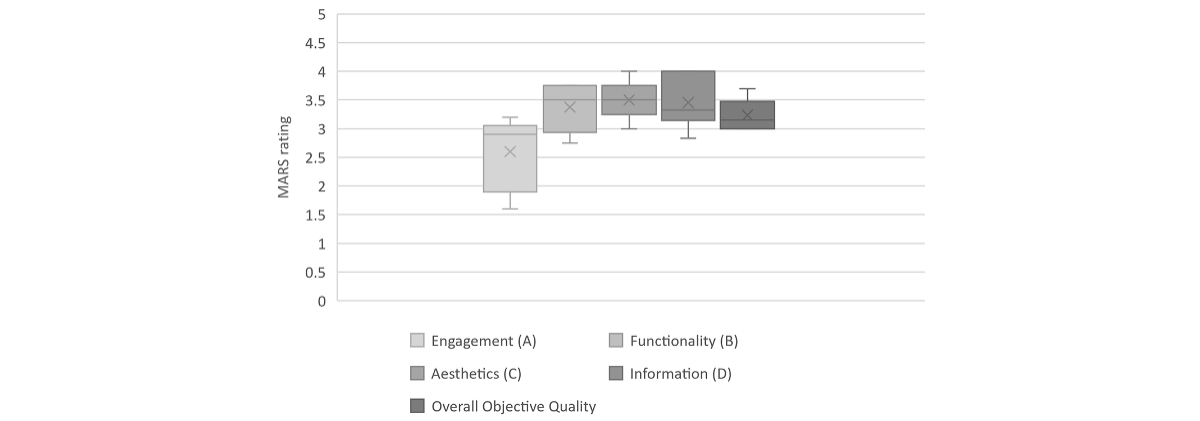
Box and whisker plot of the MARS objective quality assessments of all apps. MARS: Mobile App Rating Scale.

When considering all reviewed apps together and focusing solely on the MARS objective quality criteria, the apps showed a tendency to perform better in the (C) aesthetics category (range 3-4; mean 3.50, SD 0.35) and (D) information category (range 2.84-4; mean 3.46, SD 0.46). However, their performances in (A) engagement were much lower (range 1.6-3.2; mean 2.6, SD 0.64; Figure 2). This discrepancy may be attributed to the “customization” score under the (A) engagement category, which scored the lowest (mean 1.33, SD 0.82 out of 5). On the other hand, the “gestural design” under the (B) functionality category scored the highest (mean 4, SD 0.63 out of 5), contributing to a slightly higher overall “functionality” score.

### Accessibility Appraisal of the Included Apps: WCAG 2.1

The results from the accessibility evaluation using WCAG 2.1 are presented in Table 4. None of the reviewed apps conformed to either the A or AA version of WCAG 2.1. Conformance to these standards means that there is no content that violates the success criteria [42]. Considering that the multitude of criteria and any single issue such as a broken link or lack of voice-over recognition of a piece of text can cause an app to fail, this outcome is not surprising.

When evaluated against the overall success criteria for WCAG 2.1A, which is the most used standard in the field to meet basic accessibility requirements, apps achieved a conformance rate ranging from 65% to 86%. However, this range dropped to 53% to 71% when evaluated against WCAG 2.1AA. BEAT-D and DIYModify received the highest ratings in both evaluations (Table 4).

It is worth noting that all apps fulfilled at least 1 or 2 subcriteria of the WCAG. For example, DIYModify met all criteria under the “understandable” and “robust” categories of both the A and AA versions of WCAG 2.1. BEAT-D also met both criteria but only for WCAG 2.1A. Furthermore, all apps met the “robust” criteria of WCAG 2.1A (Table 4).

On examining the individual assessment items, we found that all apps passed at least a couple of items in each success criterion. In the “perceivable” category, all apps successfully met the assessment items of info and relationships (1.3.1) and meaningful sequence (1.3.2). Similarly, in the “operable” category, all apps passed the assessment items of 3 flashes (2.3.1) and pointer cancellation (2.5.2). Moving to the “understandable” category, the apps fulfilled the assessment items of language of page (3.1.1), language of parts (3.1.2), on focus (3.2.1), consistent navigation (3.2.3), and consistent identification (3.2.4). Lastly, in the “robust” category, the apps satisfied the assessment items of parsing (4.1.1) and name, role, value (4.1.2).

However, none of the reviewed apps managed to pass 3 assessment items. These items include resize text (1.4.4), which evaluates the ability to zoom in and enlarge text; reflow (1.4.10), which assesses the ability to reflow and adjust the content to fit the screen when zoomed in; and text spacing (1.4.12), which examines the ability to customize text characteristics. All these criteria are measured against the WCAG 2.1 AA level standards.

**Table 4:** Web Content Accessibility Guidelines (WCAG) 2.1 assessment success criteria and the assessment.

App name	Success criteria for WCAG 2.1 A					Success criteria for WCAG 2.1 AA				
	Perceivable	Operable	Understandable	Robust	Overall passing	Perceivable	Operable	Understandable	Robust	Overall passing
BEAT-D	4/5	7/9	5/5^a^	2/2^a^	0.86	6/13	9/12	9/9^a^	2/3	0.70
DC Carehomes	4/5	6/10	73/5	2/2^a^	0.68	8/13	8/13	7/9	2/3	0.66
DIYModify	6/9	6/7	4/4^a^	2/2^a^	0.82	8/17	8/9	7/7	2/2^a^	0.71
HomeFit AR	5/5^a^	4/8	2/4	2/2^a^	0.68	8/12	4/10	6/8	2/2^a^	0.63
MapIt Desktop	1/4	7/10	3/4	2/2^a^	0.65	1/11	8/13	6/7	3/3^a^	0.53
MapIt Mobile	1/4	8/8^a^	3/5	2/2^a^	0.73	4/11	8/10	6/9	2/2^a^	0.63

^a^Apps fulfilled a subcriterion of the WCAG 2.1. These items show that all apps met at least 1 or 2 subcriteria of the WCAG 2.1 despite their failure to meet WCAG 2.1 in its entirety.

### The Objective Quality and Accessibility: Visual Synthesis of MARS×WCAG

**Figure 3:**
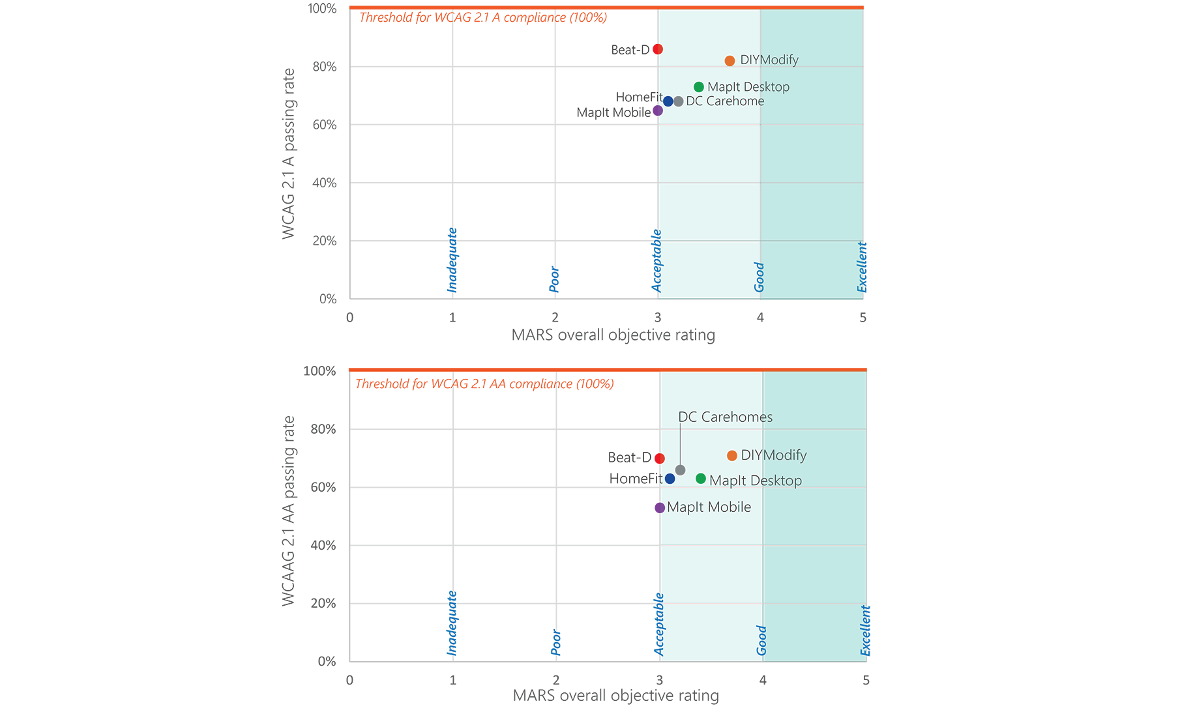
The visualization of apps’ MARS quality and accessibility using the WCAG 2.1 A and AA criteria. This visualization indicates that all reviewed apps fall within the “acceptable” range, yet they fall short of achieving the “good” range as measured by the MARS tool. None of the apps managed to meet accessibility compliance when evaluated against both WCAG 2.1 A and AA standards. The lower visualization illustrates a greater level of challenge in meeting the WCAG 2.1 AA standard, which is a more stringent criterion than WCAG 2.1 A. MARS: Mobile App Rating Scale; WCAG: Web Content Accessibility Guidelines.

The synthesis of the results from the MARS objective quality and WCAG assessments is visually represented in Figure 3. This visualization illustrates the performance of different apps based on the MARS overall objective quality appraisal and the WCAG 2.1 accessibility criteria. Although none of the apps met the thresholds to be considered both accessible and “good” according to WCAG 2.1 A and MARS, respectively, all of them were fairly close to these thresholds. Notably, DIYModify and MapIt Desktop achieved high scores for both accessibility and the MARS objective quality. While BEAT-D performed well in terms of accessibility, there is room for improvement in its overall MARS quality. On the other hand, HomeFit AR, MapIt Mobile, and DC Carehomes scored lower in both accessibility and MARS objective quality (Figure 3).

## Discussion

### Principal Findings

mHealth apps are expected to empower users with the capability to assess an individual’s functional capacities and the environmental conditions crucial for comprehensive home evaluations [43]. Key areas such as the entrance, bathroom, kitchen, and bedroom hold significant importance in home assessments, which is reflected in conventional paper-and-pencil assessment tools and should thus be integral components of the app [21,38]. Furthermore, these apps should not only offer a succinct summary of the assessment but also motivate users to take up the subsequent steps after assessment, fostering engagement among all parties involved, including health care and housing professionals, as well as consumers and their caregivers. The manner in which these apps facilitate these processes should embody comprehensiveness, engagement, and accessibility.

Our findings demonstrate that, currently, there are no apps available in the United States that meet all of these criteria. Specifically, none of the apps allowed for the assessment of functional limitations of consumers, a crucial element in identifying areas requiring assessment. The MARS ratings revealed that all the apps were near the lower threshold of the acceptable range, with the exception of DIYModify; however, none of them reached the “good” range. The low scores in the “engagement” category, in particular, need further exploration as significant factors contributing to these apps’ lower ratings. Furthermore, none of the reviewed apps met the accessibility criteria.

Despite these findings, our team notes that DIYModify scored the highest in our multidimensional assessment. We also observed that when used collectively, MapIt Mobile, MapIt Desktop, and the creator’s instruction website demonstrate strong potential. Although we assessed them separately based on the parameters of our review, we found that using the entire suite together was highly effective for visualizing multiple measurements on a 3D scan. Offering an alternative viewing option on a larger screen device could prove beneficial for apps with complex user interfaces or content. On the other hand, the other apps (HomeFit AR, BEAT-D, and DC Carehomes) were primarily questionnaire based and could have been easily accomplished without the need for an app.

### Comparison With Prior Work

Our findings align with previous meta-analyses of home assessment tools, encompassing both paper-and-pencil formats as well as technology-assisted formats [10,23]. These studies have consistently revealed a lack of comprehensive and user-friendly technology tools that can be used to assess the home environment in relation to the functional abilities of its residents. Using technology to assess home environments in order to enhance accessibility and prevent falls and other injuries has remained challenging despite the rapid advancements in 3D modeling, virtual reality, and AR over the past few decades [10,23].

Within the limited pool of tools identified in previous studies, the majority were either pilot studies or exploratory qualitative studies [10]. Only a small fraction of these tools successfully transitioned into commercially available products, as confirmed by our search process across multiple app stores. Furthermore, review studies evaluating the efficacy of home assessment tools in all formats consistently demonstrated that the traditional paper-and-pencil assessment method was more effective in identifying issues [10]. This indicates a continued preference for the traditional method over digital alternatives among occupational therapy professionals. The findings of our study, which revealed that none of the reviewed apps allowed users to assess functional limitations, are in line with these earlier observations. This limitation hampers the effectiveness of the apps in detecting problematic areas and assessing accessibility.

Our study expands on previous research by evaluating the overall quality and accessibility of mHealth home assessment tools, emphasizing their significance in promoting consumer engagement and their follow-up actions. Specifically, we found that all of the reviewed apps met the minimum acceptable quality, but none reached the threshold of “good” quality. Additionally, none of the apps met the accessibility criteria as measured by the WCAG. This is a glaring omission as the home assessment and the subsequent modification are to help aid individuals with functional limitations in their home. To facilitate the active engagement and informed decision-making of older adults and individuals with functional limitations in their health care, as well as to empower them to undertake necessary home modifications, the development of apps that prioritize engagement, activation, and accessibility becomes imperative.

We anticipate that meeting this goal will remain challenging in the foreseeable future. Developing a mobile app may seem straightforward on the surface but can quickly become a multimillion-dollar project for several reasons. First, the development of mHealth apps for home assessment, with a focus on reliability, precision, and user-friendliness, necessitates robust interdisciplinary collaboration involving occupational therapists, building professionals, and user interface and user experience design experts.

Second, the inherent limitations in precision with current 3D scanning and AR technology, along with the need to meet the requirements of various devices, quickly add another layer of complexity to the endeavor [44-47]. Third, creating user-friendly apps requires extensive usability testing across diverse populations. In the case of home assessment, this involves testing with individuals exhibiting various functional limitations and their caregivers, contributing to the overall complexities and high cost of conducting such studies.

In contrast, the current funding landscape shows a tendency to prioritize research emphasizing cutting-edge scientific discovery or direct health outcomes with large-scale clinical trials. While the benefits of home modification have been studied either through indirect measures such as falls and emergency department visits via secondary data analysis [48,49], or a clinical trial [50], measuring the direct health outcome of the home assessment itself remains rather obscure.

Additionally, the transition from discovery to commercialization, as discussed earlier, introduces additional intricacies. Deploying the app in the market sustainably necessitates ongoing support, updates, and maintenance, contributing to the long-term cost of app development, which academic endeavors are not well suited for, often impeding the provision of free or affordable consumer apps. Above all, the lack of awareness and appreciation of the benefits that come from home assessments and home modification in the general public appears to be a key hindrance [11,12], discouraging adequate investment in this critical domain. With the rapid aging of the population and increasing interest and awareness from both the consumer market and the government alike, we hope that adequate resources are invested, fostering innovations in academia and the commercial realm alike.

### Strengths and Limitations

Our review boasts several strengths. First, our comprehensive search encompassed scholarly databases, the US Google Play Store, Apple App Store, and fnd.io, ensuring a thorough exploration of available resources. The convergence of these searches instilled confidence in the thoroughness of our efforts. Furthermore, the significant disparity between the results of the scholarly database search and the app store search shed light on the challenges associated with translating scholarly endeavors into practical applications through the commercialization process.

Another strength lies in the complementary use of multiple rigorous assessments, focusing on both quality and accessibility. For instance, the BEAT-D app achieved a high passing percentage for WCAG criteria, yet it ranked lower in the MARS evaluation. On the other hand, MapIt Desktop had a lower score in WCAG, but ranked higher in the MARS. This discrepancy emphasizes the importance of conducting both assessments, particularly in the context of home assessment tools where individuals with functional limitations play a crucial role.

Finally, while evaluating apps with the MARS assessment is standardized and relatively straightforward, assessing apps based on WCAG criteria requires a more substantial time investment to grasp each of the 50 criteria, which is extensively explained on the WCAG’s website. To facilitate the use of WCAG, our research team has developed an evaluation form that includes concise summaries of each criterion, which can be used in future studies, streamlining the evaluation process (Multimedia Appendix 1).

The review also presents certain weaknesses that deserve attention. First, despite our efforts to conduct a comprehensive search for all available home assessment tools, the inclusion of apps was limited by their availability in the US market. It is worth highlighting that several apps, discovered via our database search, fnd.io search, and personal connections, demonstrate promise but remain unavailable on US app stores.

Second, our testing of multiplatform apps was focused solely on the iPhone versions of all reviewed apps. This limitation was observed during the assessment of WCAG criterion 1.3.4 (orientation), which assesses the device’s capacity to transition between portrait and landscape modes. Notably, the iPhone variant of BEAT-D did not meet this criterion, while the iPad version might have passed had it been evaluated. To ensure a more comprehensive assessment, it would be advantageous to test these apps on all compatible devices, including those running on Android operating systems.

Finally, while assessing apps with the MARS and WCAG provided valid insights into the overall quality and accessibility of apps based on established criteria, future studies will have to take into consideration consumer-level feedback, particularly focusing on the firsthand experiences of those with various functional capacities and their caregivers.

### Conclusions

A proficient home assessment tool, designed to engage consumers, health care providers, and housing professionals, should offer reasonable functionality and possess objective quality and accessibility. However, our findings bring to light that none of the currently available home assessment mHealth apps in the United States align with these benchmarks. None of the apps offered sufficient methods to assess individuals’ functional capacity and conduct comprehensive environmental assessments, and they fell short of meeting the WCAG accessibility criteria. Furthermore, although every app reached an “acceptable” level, none of them attained a “good” level in the MARS quality evaluations.

## Appendix

Multimedia Appendix 1 PRISMA (Preferred Reporting Items for Systematic Reviews and Meta-Analyses) 2020 checklist.

Multimedia Appendix 2 PRISMA (Preferred Reporting Items for Systematic Reviews and Meta-Analyses) 2020 for abstracts checklist.

Multimedia Appendix 3 Accessibility evaluation form adapted from Web Content Accessibility Guidelines (WCAG) 2.1.

## Abbreviations

AR: augmented reality
HE: Housing Enabler
I-HOPE: In-Home Occupational Performance Evaluation
MARS: Mobile Application Rating System
mHealth: mobile health
NA: not applicable
PRISMA: Preferred Reporting Items for Systematic Reviews and Meta-Analyses
WCAG: Web Content Accessibility Guidelines
