# Supplementary material for: Quality and Accessibility of Home Assessment mHealth Apps for Community Living: Systematic Review
Source: JMIR Mhealth Uhealth. 2024 Mar 11;12:e52996. doi: 10.2196/52996 (PMC10980499; doi:10.2196/52996)
Supplement: Multimedia Appendix 3 [file mhealth_v12i1e52996_app3.doc]

Multimedia Appendix 1. Accessibility evaluation form adapted from WCAG 2.1.

| **WCAG App Evaluation Form** | | | | | | | |
| --- | --- | --- | --- | --- | --- | --- | --- |
| App Name: | | | | | | | |
| Reviewer: | | | | | | | |
| **Category** | **Number** | **Version** | **Level** | **Success Criterion** | **Summary Description** | **Pass/Fail/NA** | **Notes** |
| PERCEIVABLE | 1.1.1 | 2.0 | A | Non-text Content | Every piece of non-text content is accompanied by a text alternative. Symbols, icons, and images are required to have corresponding text, except for decorative ones. If assistive technology can vocalize the symbol, icon, or image, there's no need for additional text. |  |  |
|  | 1.2.1 | 2.0 | A | Audio-only and Video-only (prerecorded) | Equivalent alternatives are provided for pre-recorded audio or video content. |  |  |
|  | 1.2.2 | 2.0 | A | Captions (prerecorded) | All synchronized media, which is a combination of video and audio, come with accompanying captions. |  |  |
|  | 1.2.3 | 2.0 | A | Audio Description or Media Alternative (prerecorded) | For all synchronized media, which refers to the combination of video and audio, an audio description **or** a text alternative is offered. Audio descriptions provide further context to a video, extending beyond just the dialogue. |  |  |
|  | 1.2.4 | 2.0 | AA | Captions (Live) | All live content is accompanied by captions. |  |  |
|  | 1.2.5 | 2.0 | AA | Audio Description (Prerecorded) | For pre-recorded synchronized media, which combines audio and video, an audio description is available. Such descriptions offer further insights into the video content, going beyond just the dialogue. |  |  |
|  | 1.3.1 | 2.0 | A | Info and Relationships | The information, structure, and relationships expressed through presentation are also accessible in text form. |  |  |
|  | 1.3.2 | 2.0 | A | Meaningful Sequence | When the meaning of the text depends on its order, it can be programmatically determined. **Suggested Assessment Tool: VoiceOver** |  |  |
|  | 1.3.3 | 2.0 | A | Sensory Characteristics | The operation of content is not exclusively dependent on sensory characteristics like shape, color, size, sound, and so on. |  |  |
|  | 1.3.4 | 2.1 | AA | Orientation | The content is viewable in both portrait and landscape orientations. |  |  |
|  | 1.3.5 | 2.1 | AA | Identify Input Purpose | Input fields serving standard functions can be programmatically discerned. Assistive technology should be able to detect a text field and vocalize its purpose if it aligns with standard types such as name, birthdate, username, etc.  **Suggested Assessment Tool: VoiceOver** |  |  |
|  | 1.4.1 | 2.0 | A | Use of Color | Information is not conveyed solely through the use of color. |  |  |
|  | 1.4.2 | 2.0 | A | Audio Control | Users have the option to halt audio playback. |  |  |
|  | 1.4.3 | 2.0 | AA | Contrast (Minimum) | Small text features a contrast ratio of 4.5:1 against the background, while large text displays a ratio of 3:1. This does not apply to decorative elements and only pertains to text smaller than 18pt in size, or below 14pt if the font is bold.  **Suggested Assessment Tool: WCAG Contrast Checker** |  |  |
|  | 1.4.4 | 2.0 | AA | Resize Text | In the app, text can be magnified up to 200 percent without the need for assistive technology. On a mobile device, this includes the ability to zoom in using finger gestures. |  |  |
|  | 1.4.5 | 2.0 | AA | Images of Text | Text should be in a format that can be detected by assistive technology and not embedded within an image.  **Suggested Assessment Tool: VoiceOver** |  |  |
|  | 1.4.10 | 2.1 | AA | Reflow | When magnified to 200%, the text should reflow so that scrolling in two directions is not necessary. It is likely that finger zooming will not be effective in this case. |  |  |
|  | 1.4.11 | 2.1 | AA | Non-text Contrast | Graphical controls, including buttons and text input boxes, require a contrast ratio of 3:1. Consider whether the graphical object remains comprehensible if the color in question is made invisible. |  |  |
|  | 1.4.12 | 2.1 | AA | Text Spacing | The spacing of the text is customizable. The line height (or line spacing) should be at least 1.5 times the font size. The spacing after paragraphs should be at least twice the font size. Letter spacing (or tracking) should be at least 0.12 times the font size. Word spacing should be at least 0.16 times the font size. |  |  |
|  | 1.4.13 | 2.1 | AA | Content on Hover or Focus | For content that becomes visible when hovered over, the following should apply: (1) users should have the option to turn it off, (2) the cursor should be able to traverse the content without causing it to vanish, and (3) the extra content should stay visible until the hover action is no longer active.  **Does Not Apply to Mobile** |  |  |
| OPERABLE | 2.1.1 | 2.0 | A | Keyboard | All content is accessible and operable using a keyboard interface. You can enable this by connecting a keyboard via Bluetooth and turning on the full keyboard access feature.  **Suggested Assessment Tool: Bluetooth Keyboard** |  |  |
|  | 2.1.2 | 2.0 | A | No Keyboard Trap | A user is not confined within keyboard input. This implies that you can use the 'tab' key to exit the text box and cease typing.  **Suggested Assessment Tool: Bluetooth Keyboard** |  |  |
|  | 2.1.4 | 2.1 | A | Character Key Shortcuts | If there are keyboard shortcuts that use only a single letter, punctuation mark, number, or symbol, then at least one of the following must hold true: the shortcut can be deactivated, the shortcuts can be customized, or the shortcut only becomes active when the corresponding component is in focus.  **Suggested Assessment Tool: Bluetooth Keyboard** |  |  |
|  | 2.2.1 | 2.0 | A | Timing Adjustable | In cases where a time limit is imposed, it is essential to provide users with the ability to request additional time or disable the time limit altogether. |  |  |
|  | 2.2.2 | 2.0 | A | Pause, Stop, Hide | Users should have the capability to pause, stop, or hide any content that involves movement, blinking, scrolling, or automatic updates. |  |  |
|  | 2.3.1 | 2.0 | A | Three Flashes or Below Threshold | Content should not contain more than three flashes within a one-second period. |  |  |
|  | 2.4.1 | 2.0 | A | Bypass Blocks | Users have the ability to skip over repeated content. |  |  |
|  | 2.4.2 | 2.0 | A | Page Titled | Pages must have titles. |  |  |
|  | 2.4.3 | 2.0 | A | Focus Order | The focus order, determined by tabbing, should follow a logical navigation sequence.  **Suggested Assessment Tool: Bluetooth Keyboard** |  |  |
|  | 2.4.4 | 2.0 | A | Link Purpose (In Context) | The purpose of a link can be discerned solely from its link text. |  |  |
|  | 2.4.5 | 2.0 | AA | Multiple Ways | There should be a means to access the content in a more convenient manner, such as through a table of contents. |  |  |
|  | 2.4.6 | 2.0 | AA | Headings and Labels | The headers and labels are descriptive in nature. |  |  |
|  | 2.4.7 | 2.0 | AA | Focus Visible | The method used to indicate keyboard input is noticeable. |  |  |
|  | 2.5.1 | 2.1 | A | Pointer Gestures | All features and functionalities that depend on multipoint or path-based gestures should also be operable using a single pointer, unless such gestures are essential, such as in the case of drawing a free-form shape. Complex gestures like dragging, pinching, or performing a two-finger swipe may not meet this requirement. A single pointer refers to a single click or tap. |  |  |
|  | 2.5.2 | 2.1 | A | Pointer Cancellation | For functions that necessitate a single click or tap, the operation should be executed upon releasing the button or lifting the finger, rather than during the initial press or touch. |  |  |
|  | 2.5.3 | 2.1 | A | Label in Name | The text spoken by assistive technology should match the words displayed visually.  **Suggested Assessment Tool: VoiceOver** |  |  |
|  | 2.5.4 | 2.1 | A | Motion Actuation | Functions that typically involve moving a device (such as shaking or tilting) or gesturing towards the device should also offer alternative methods for completion. |  |  |
| UNDERSTANDABLE | 3.1.1 | 2.0 | A | Language of Page | Assistive technologies have the ability to detect the primary language of the content, such as English.  **Suggested Assessment Tool: VoiceOver** |  |  |
|  | 3.1.2 | 2.0 | AA | Language of Parts | Assistive technologies have the ability to detect a change in language, such as the use of a French word.  **Suggested Assessment Tool: VoiceOver** |  |  |
|  | 3.2.1 | 2.0 | A | On Focus | A new window should not open, and a change of context should not occur unless it is necessary. |  |  |
|  | 3.2.2 | 2.0 | A | On Input | When there is user input involved, it is important to inform the user in advance before a change of context occurs. For instance, when answering a question a user should click a submit button before being directed to the next page. |  |  |
|  | 3.2.3 | 2.0 | AA | Consistent Navigation | Repeated navigational mechanisms should appear in the same relative order throughout the content. |  |  |
|  | 3.2.4 | 2.0 | AA | Consistent Identification | Components that have the same functionality are identified and operate consistently. |  |  |
|  | 3.3.1 | 2.0 | A | Error Identification | When an input error is detected automatically, it is communicated to the user as an error. |  |  |
|  | 3.3.2 | 2.0 | A | Labels or Instructions | When user input is necessary, labels or instructions are provided. |  |  |
|  | 3.3.3 | 2.0 | AA | Error Suggestion | When an input error is automatically detected, the system provides suggestions to help the user correct them. |  |  |
|  | 3.3.4 | 2.0 | AA | Error Prevention (Legal, Financial, Data) | If legal commitments or financial transactions are involved in the content, the submission process allows for reversibility or the data is thoroughly checked, giving the user an opportunity to correct any errors. |  |  |
| ROBUST | 4.1.1 | 2.0 | A | Parsing | Readable via assistive technology.  **Suggested Assessment Tool: VoiceOver** |  |  |
|  | 4.1.2 | 2.0 | A | Name, Role, Value | Readable via assistive technology.  **Suggested Assessment Tool: VoiceOver** |  |  |
|  | 4.1.3 | 2.1 | AA | Status Messages | Assistive technologies have the capability to present status messages such as "invalid entry."  **Suggested Assessment Tool: VoiceOver** |  |  |
|  |  |  |  |  | **TOTALS: *exclude NA** | ex: 9/10 |  |
|  |  |  |  |  | Perceivable |  |  |
|  |  |  |  |  | Operable |  |  |
|  |  |  |  |  | Understandable |  |  |
|  |  |  |  |  | Robust |  |  |
|  |  |  |  |  | Composite |  |  |
